# 白血病异基因造血干细胞移植后HLA区域杂合性缺失的研究进展

**DOI:** 10.3760/cma.j.issn.0253-2727.2022.07.015

**Published:** 2022-07

**Authors:** 铭浩 林, 明瑞 霍, 翔宇 赵

**Affiliations:** 北京大学人民医院、北京大学血液病研究所，国家血液系统疾病临床医学研究中心，北京 100044 Peking University People's Hospital, Peking University Institute of Hematology, National Clinical Research Center for Blood Diseases, Beijing 100044, China

异基因造血干细胞移植（allo-HSCT）是治疗多种血液系统恶性肿瘤，尤其是白血病的重要手段。经allo-HSCT治疗后，白血病细胞的清除可通过供者淋巴细胞输注（DLI）完成，这是一种过继免疫疗法，可将正常供者来源的外周血淋巴细胞输注到患者体内以诱导移植物抗肿瘤（GVT）效应，继而促进移植后免疫系统的快速重建，清除患者体内残留的癌细胞[1]。随着预处理方案、移植物抗宿主病（GVHD）预防和支持治疗的改进，白血病移植相关死亡率得到有效降低，但肿瘤复发依然是allo-HSCT后死亡的首要原因。在恶性肿瘤中，染色体杂合性缺失（LOH）是一种常见的染色体畸变方式，表现为同源染色体上一个等位基因的部分或全部遗传物质发生丢失。研究表明，白血病复发的因素多样，人类白细胞抗原（HLA）杂合性缺失（简称为HLA loss）是其中一个重要的诱因。本文就HLA loss的发生发展、检测及其潜在的临床治疗策略的研究进展进行综述。

一、HLA概况

主要组织相容性复合体（MHC）是一组存在于脊椎动物中的紧密连锁的基因家族，可编码生物相容复合体抗原，与免疫调节、免疫应答及移植排斥等密切相关。人类的MHC称为HLA，负责编码HLA抗原或HLA分子。HLA基因复合体定位于人类第6号染色体短臂6p21.31，全长约3.6 Mb，包括HLA Ⅰ类、Ⅱ类和Ⅲ类基因区。经典的HLA Ⅰ类基因和Ⅱ类基因所编码的产物在组织分布、结构和功能上各有特点，显示出极为丰富的多态性，具有抗原提呈功能，能直接参与T细胞的激活和分化。同时，作为主要移植抗原，HLA可在同种异体移植中引起移植排斥反应。而非经典HLA分子主要参与调控固有免疫应答或参与抗原加工，仅表现出有限的多态性。HLA基因的遗传特点包括多态性、单体型和连锁不平衡，在遗传过程中，HLA单倍型作为一个遗传单位直接传给子代[2]，这正是单倍型造血干细胞移植（haplo-HSCT）的理论基础。

二、HLA loss的研究进展

（一）HLA loss的发生率

allo-HSCT在血液系统疾病的治疗应用中已取得了长足的进展，但移植后复发仍是影响造血干细胞移植患者预后的主要原因之一。研究表明，allo-HSCT后有20％～25％的急性髓系白血病（AML）患者会发生疾病复发，而高危AML患者的复发率可达30％～50％[3]。移植后复发的白血病患者预后较差[4]，国际骨髓移植登记组（CIBMTR）的资料显示，2008至2018年间，疾病复发是allo-HSCT的最常见死因，在移植后100 d内和100 d后的死因中分别占27％、61％[5]，这可能与HLA基因的异常改变相关。HLA分子的突变、下调或基因组的丢失在实体肿瘤中较为常见，McGranahan等[6]就曾报道40％的非小细胞性肺癌存在HLA loss，从而引起免疫逃逸。2009年，Vago等[7]首次发现HLA loss可导致AML患者haplo-HSCT后复发。研究认为，残留的白血病细胞可以通过基因组丢失不相合的HLA来逃避同种异基因供者淋巴细胞的清除。

现阶段，由于针对白血病患者HLA基因的检测方法尚未普及，对HLA loss发生率的研究并不多。Crucitti等[8]研究发现HLA loss型复发在haplo-HSCT后的发生率最高，约占复发疾病的33％。全球的一项多中心移植后复发队列研究小组报道了在不同移植模式下HLA loss复发型的发生率，在396例血液系统疾病复发患者中，HLA loss发生率在haplo-HSCT、不匹配非亲缘供者造血干细胞移植、HLA相合非亲缘供者造血干细胞移植中分别占22.6％、11.9％和4.3％，而在脐血移植中尚未发现HLA loss型复发[9]。随着对HLA loss检测的重视度逐渐增加，我国也报告了多例HLA loss型白血病复发患者[10]–[11]。

（二）HLA loss的发生机制及其临床后果

染色体杂合性缺失是白血病患者中一种常见的染色体畸形。以haplo-HSCT为例，在haplo-HSCT移植环境中，患者和供者之间存在HLA单倍型不相合。基于此，一方面，通过对白血病细胞表面HLA分子的识别，输注的供者淋巴细胞可介导对残余白血病细胞的清除。另一方面，在这种强烈的免疫应激状态下，肿瘤细胞HLA基因可能发生获得性单亲二倍体（aUPD）而导致HLA错配基因缺失。Vago等[7]在分析43例接受haplo-HSCT的AML和骨髓异常增生综合征（MDS）患者时，发现有17例复发，其中5例复发患者复发白血病细胞的免疫表型和细胞遗传学特征与初诊时相同，且并未观察到新的细胞遗传学异常。随后，他们利用全基因组单核苷酸多态性（SNP）阵列分析对这一现象进行更深入的研究，发现这5例复发患者的染色体6p存在拷贝数中性的杂合性缺失（CN-LOH），即发生HLA loss。研究认为，在免疫应激和基因组不稳定等因素作用下，不相合的HLA基因区域可发生同源重组，被替换成与供者相合的HLA，导致该区域获得纯合性，而不会实际丢失基因组物质。值得一提的是，发生杂合性缺失的HLA基因区域在人群中并非固定不变，但在大多数情况下，通常包括整个HLA区域，即包括所有HLA Ⅰ类和Ⅱ类区域。

之后，其他一些研究也陆续报道了allo-HSCT后HLA loss型白血病复发的病例[8],[12]–[13]。Crucitti等[8]发现HLA loss的发生率与移植时输注的T细胞数量直接相关，而T细胞数量在一定程度代表免疫应激的程度，这表明HLA loss的发生与免疫应激相关。不过，在“T细胞裸露”的移植环境下，HLA loss仍可能发生，提示HLA loss可能与多种因素相关。后续的研究也尝试去解释不同移植环境下HLA loss发生率的差异现象，如针对haplo-HSCT的HLA loss发生率远高于无关供者造血干细胞移植的现象，研究认为可从两个角度进行分析：①当供受者的HLA不相容较少时，供者T细胞同种异体反应性和GVL效应可能更多地通过对次要组织相容性抗原识别来实现，因而对HLA基因组的免疫压力也相对降低。②在不相关的造血干细胞移植中，HLA不相合区域并不完全定位在同一单倍型染色体上，这意味着通过aUPD丢失一个不匹配的单倍型在减轻免疫压力方面可能不如在haplo-HSCT中有效。然而，不同HLA位点的错配在驱动同种异体反应和GVL效应方面的相对贡献尚不完全清楚。

研究HLA loss能否发生在allo-HSCT前对于了解白血病复发的机制以及避免因HLA配型错误影响移植效果非常重要。到目前为止，科学家对aUPD的分子驱动因素了解还不够深入。不过已有研究证明，染色体断裂易感因素的暴露和DNA损伤诱导剂的作用，如一些化疗药物的影响，可能会导致紧密靠近有丝分裂重组位点的aUPD风险增加[14]。2008年，一项纳入454例AML患者的研究指出，allo-HSCT前AML患者体内基因发生aUPD的概率为15％～20％，这种改变主要影响特定的染色体臂（如13q、11p和11q），不过当时仅报告了1例涉及6p21的aUPD现象[15]。赵佳炜等[16]通过HLA基因分型和家系分析方法也报道了1例急性混合细胞白血病患者初诊时发生HLA loss的现象。随着进一步的研究，Dubois等[17]认为AML患者HLA loss的发生率不低于3％～4％。尽管白血病患者移植前HLA loss的发生不是偶然事件，但常规HLA配型检测难度仍较高，需要患者初诊和缓解期的HLA分型结果以及家系HLA相关数据的支持。

Horowitz等[18]发现，HLA loss复发型白血病患者的复发时间大多较晚（中位复发时间为移植后307 d），远晚于非HLA loss型（中位复发时间为移植后88 d）。这也从另一个角度印证了HLA loss多发生于移植后，若为原本存在的aUPD引起的HLA基因异常，残留的白血病细胞克服免疫控制较为容易，应能更早引起复发。不过，HLA loss复发型白血病患者与非HLA loss复发型患者的整体存活率并无显著差异。综合考虑，有研究建议对于接受造血干细胞移植患者不应该过早放松临床随访，最好应谨慎观察至移植后第2年[8]。

总之，HLA是供者淋巴细胞发挥GVL效应最主要的免疫靶点。因此，发生HLA loss的肿瘤细胞可逃脱供者同种反应性淋巴细胞的识别及清除而进行选择性增殖，经过长期的免疫选择，可引起白血病的复发。并且，在这种复发下，此前输注的供者淋巴细胞反而可能会增加GVHD的风险，危害患者健康。

（三）HLA loss的检测

HLA loss复发型白血病的高发生率和不良预后体现了HLA loss检测的必要性。不过，现阶段尚没有检测白血病细胞中HLA loss的标准化方法。目前，主要有两种HLA loss的检测策略：①对复发后HLA基因丢失的检测可基于患者STR嵌合率与HLA嵌合率的比较分析，实时荧光定量PCR（qPCR）检测法是其中一项重要的检测手段。qPCR需要事先设计针对患者特异型HLA位点的引物探针，以检测移植后患者特异型HLA嵌合率，并通过与STR嵌合率进行对比分析来判断患者HLA loss的发生情况。这种检测方法成本低、报告快，但事先必须有患者特异性HLA位点可供检测。基于此原理开发的“HLA-KMR”诊断工具提供了一种在早期阶段检测HLA丢失复发事件的灵敏方法，有利于临床决策的快速作出，不过该工具探针只能检测HLA-A、HLA-C和HLA-DPB1等位基因位点，不能覆盖所有人群[19]。②通过二代测序法对HLA区域进行测序从而检测HLA loss的发生情况，可对全部HLA区域进行检测，从而涵盖所有的患者，但其灵敏度稍低，且时间及经济成本较高[20]。

（四）HLA loss复发型白血病的治疗策略

allo-HSCT后白血病复发的最佳治疗方法未有定论，HLA loss复发型白血病患者的临床治疗方案仍处在探索阶段。

1. 二次移植：由于HLA loss型复发的特殊性，2019年，HLA loss的检测已被纳入欧洲血液与骨髓移植学会（EBMT）《HLA单倍型供者的供者淋巴细胞输注的临床应用共识》，共识中指出，对于确诊的HLA loss患者，推荐使用不同的单倍型供者或无关供者，而原供者淋巴细胞输注不能获益[21]。换句话说，二次移植应选择选取与患者未丢失HLA单倍型错配的供者[22]。以HLA单倍体整条丢失为例，假设患者正常细胞HLA单倍型为A1/A2，经第一次haplo-HSCT和DLI治疗（选用A1/A3型细胞）后，患者发生了HLA loss，导致肿瘤细胞HLA表型改变为A1/A1，而体细胞依然为A1/A2，此时在第二次移植时应选用A2/A3型供者细胞进行移植。通过这种途径，二次移植的供者淋巴细胞能重新识别肿瘤细胞的不相容HLA抗原，从而再次具备识别和杀死白血病细胞的能力，并且也能降低患者GVHD的风险。Imus等[23]进行的研究也验证了这种治疗策略的正确性。该团队对40例于2005至2015年间因HLA loss导致AML复发的患者接受二次移植后的存活率进行研究，发现二次移植时选择不同单倍型移植的患者的总存活率显著高于接受相同供者的患者，表现为更高的移植后复发率和GVHD风险。

2. 输注非HLA限制性的供者NK细胞：不同于T/B淋巴细胞，NK细胞表面表达一系列与其活化和抑制相关的调节性受体，从而发挥其效应功能。其中，NK细胞表面杀伤性免疫球蛋白受体（KIR）含有能与HLA Ⅰ类分子结合的杀伤抑制受体结构域，从而获得免疫耐受[24]。不过，HLA loss型复发的白血病患者由于发生CN-LOH，并不会降低白血病细胞表面HLA Ⅰ类分子的整体水平，从而避免触发NK细胞的“丢失自我”杀伤机制，但白血病细胞丢失的HLA等位基因通常也包含供者抑制KIR的配体，因此NK细胞具有杀伤复发肿瘤细胞的潜力[25]。Takahashi等[26]通过体外实验发现供者NK细胞输注可有效杀伤HLA loss型肿瘤细胞。尽管如此，体外输注NK细胞仍不足以避免临床疾病的复发，这可能与复发后白血病细胞的增殖能力强和移植患者所建立的环境耐受相关[22]。不过，注入大量纯化供者NK细胞以治疗HLA loss型复发白血病的可能性仍值得研究。截至目前，移植患者NK细胞输液的临床经验有限，尚需要系统的研究。

3. 其他治疗：HLA loss其他治疗策略包括嵌合抗原受体（CAR）修饰的T细胞（CAR-T）疗法、双特异性抗体治疗等[11],[14],[27]。CAR-T疗法是利用基因改造技术在分离的T细胞表面表达抗原特异性CAR，并连接于T细胞活化基序，使得T细胞具备良好的靶向性，而无需依赖于对HLA分子的识别，是一个很有希望的治疗选择[14]。双特异性抗体治疗可使免疫细胞重新定向到恶性肿瘤，介导清除。已有研究揭示了CD3/CD33双抗治疗HLA loss复发型白血病的潜力，人体外与动物实验已表明，CD3/CD33双抗可诱导T细胞活化，靶向杀伤肿瘤细胞[27]。

三、结语与展望

综上所述，HLA loss是allo-HSCT后导致白血病复发的一种可能机制，HLA在强烈的免疫压力的情况下可能发生基因组的杂合性缺失，使得供者淋巴细胞无法对进行肿瘤细胞进行有效识别，进而躲避供者淋巴细胞的GVL效应并导致供者淋巴细胞输注无效。鉴于HLA loss复发型的高比例，建议将HLA loss诊断作为移植后有复发迹象时的常规检测项目，以更好地指导对疾病复发的干预治疗。

然而，现阶段我们对HLA loss的了解仍不够清晰。未来的研究重点应包括：①进一步探究HLA loss在不同类型白血病和移植模式中的发生情况及其机制，探寻其发生的危险因素；②开展HLA loss与白血病复发关系的前瞻性研究；③开发一种灵敏度高、操作快速的HLA loss检测方法，规范检验方案；④制定HLA loss复发型白血病的标准治疗方案，改善患者的预后。总之，相信随着对HLA loss的深入探索，我们将能更好地应对造血干细胞移植后白血病复发。
